# Advancements in Clear Aligner Fabrication: A Comprehensive Review of Direct-3D Printing Technologies

**DOI:** 10.3390/polym16030371

**Published:** 2024-01-29

**Authors:** Poom Narongdej, Mana Hassanpour, Nicolas Alterman, Frederick Rawlins-Buchanan, Ehsan Barjasteh

**Affiliations:** 1 Department of Mechanical and Aerospace Engineering, California State University Long Beach, Long Beach, CA 90840, USA; poom.narongdej@csulb.edu (P.N.); nicolas.alterman@student.csulb.edu (N.A.); 2 Institute of Mathematical Sciences, Claremont Graduate University, Claremont, CA 91711, USA; 3 Department of Chemical Engineering, California State University Long Beach, Long Beach, CA 90840, USA; mana.hassanpour01@student.csulb.edu (M.H.);

**Keywords:** 3D printing, orthodontic treatment, aligners, essential properties, challenges, narrative review

## Abstract

Clear aligners have revolutionized orthodontic treatment by offering an esthetically driven treatment modality to patients of all ages. Over the past two decades, aligners have been used to treat malocclusions in millions of patients worldwide. The inception of aligner therapy goes back to the 1940s, yet the protocols to fabricate aligners have been continuously evolved. CAD/CAM driven protocol was the latest approach which drastically changed the scalability of aligner fabrication—i.e., aligner mass production manufacturing. 3D printing technology has been adopted in various sectors including dentistry mostly because of the ability to create complex geometric structures at high accuracy while reducing labor and material costs—for the most part. The integration of 3D printing in dentistry has been across, starting in orthodontics and oral surgery and expanding in periodontics, prosthodontics, and oral implantology. Continuous progress in material development has led to improved mechanical properties, biocompatibility, and overall quality of aligners. Consequently, aligners have become less invasive, more cost-effective, and deliver outcomes comparable to existing treatment options. The promise of 3D printed aligners lies in their ability to treat malocclusions effectively while providing esthetic benefits to patients by remaining virtually invisible throughout the treatment process. Herein, this review aims to provide a comprehensive summary of studies regarding direct-3D printing of clear aligners up to the present, outlining all essential properties required in 3D-printed clear aligners and the challenges that need to be addressed. Additionally, the review proposes implementation methods to further enhance the effectiveness of the treatment outcome.

## 1. Introduction

Clear Aligners have been utilized as an alternative to orthodontic braces due to their low impact on quality of life, relative effectiveness for low-grade malocclusions, and individualized build to any patient. Manufacturing techniques have evolved since aligner’s inception to include vacuum forming, plaster molding, and more recently 3D printing. 3D printing has advantages when compared to conventional techniques, including manufacturing speed, improved patient fit, and less invasive modeling techniques, as well as the integration of more advanced technologies. With new methods, like 3D printing, comes a new set of challenges. Since new materials are necessary, their design and safety must be investigated. Common techniques used to print aligners include Digital Light Processing (DLP), Stereolithography (SLA), Polyjet (PJ), and other niche Vat Polymerization (VP) techniques to alter the chemical structures of the printable materials in unique ways, where safety and strength are paramount. The purpose of this review is to clarify the processes, advantages, and intricacies in developing 3D printable aligners as well as discuss the future of 3D printing in the dental field. In researching for this review, the authors focused on papers relating to the direct 3D printing of clear aligners published between 2017 and 2023.

## 2. 3D Printing in Dentistry

Additive manufacturing (AM), also known as 3D printing, is a process of producing physical 3D objects from digital files, format, via joining, bonding, or polymerizing materials [1,2,3,4,5]. The first 3D printing technology was invented by Charles Hull in the 1980s, which was called ‘stereolithography (SLA)’, a photopolymerization process [1,4,6]. 3D printers differ from conventional formative and subtractive manufacturing as an object is printed along the x-y plane according to the CAD file and is then built vertically along the z-axis in a layer-by-layer sequence [2,7,8]. 3D printing has gained much attention in commercial and professional industries due to its excellence in precision, material saving, freedom of design, and customization [1]. Materials that are usually used in 3D printing include metals, ceramics, and polymers [2,9,10]. Furthermore, research has been done in identifying dental resins to be used in the clinical setting, such as Bis-EMA, UDMA, TEGDMA, and Bis-GMA, by combining monomers like PMMA with oligomers, photoinitiators, and sometimes nanoparticles [11,12,13,14,15,16].

3D printing was first introduced in the dental field in the early 2000s to fabricate implants and custom prosthetics. Combining 3D printing technology with 3D visual modeling led to a viable and user-friendly technique that aided dentists in diagnostics and repair [1]. The 3D printing process in dentistry can be categorized by five major steps: (1) 3D model data acquisition, (2) design STL file development, (3) Model preparation for printing (slicing), (4) model printing, and (5) post-processing [2,3,17,18,19,20].

## 3. Advantages of 3D Printing

3D printing allows dentists to fabricate very specific appliances and scaffolds that are difficult with conventional methods while also providing the ability to do so in-office. This leads to the treatment of more patients per doctor and allows more control over their treatment plans, providing patients with an efficient, safe, and more comfortable experience [2,21]. The greatest advantage of 3D printing is that it allows medical products and equipment to be freely customized and individualized for each patient [1,7]. The emergence and development of additive technologies brought with it advancements in high-resolution and complex geometrical dentures (e.g., aligners, inlays, onlays, and coverage crowns) that can be produced with various materials, and at a higher efficiency [1,10,17,22]. This technology gives the ability to make personalized distalizers less complicated, more accurate, and have shorter cycle times [17,21]. In addition, due to the low material waste and labor costs, 3D printing is great for personalized and small-scale productions that may be unreasonable with conventional, large-scale techniques [4,7,23].

## 4. 3D Printing Methods in Dentistry

**Figure 1:**
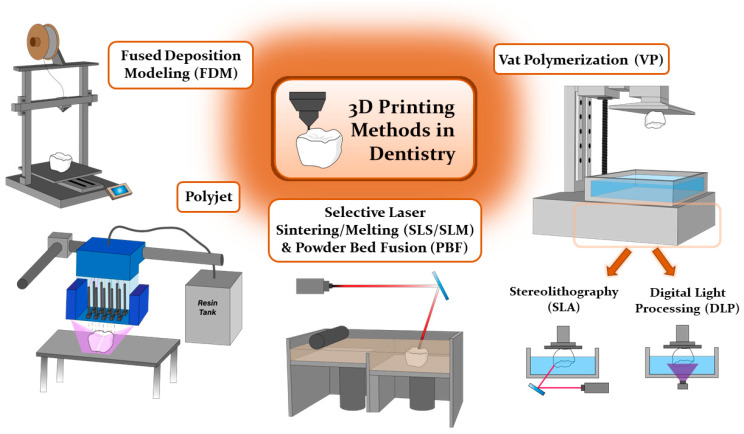
Different 3D printing techniques in dentistry. Adapted from [1,4,7,24].

Current digital manufacturing can be broadly classified into two categories: (1) subtractive manufacturing (SM) and (2) additive manufacturing (AM). In subtractive manufacturing, dental dentures are created by mechanically cutting the material, often referred to as milling, to achieve the desired geometrical configurations. While this method offers the advantage of reduced manufacturing time, it is associated with significant material wastage [2,22]. Aligners are primarily produced using additive manufacturing techniques like Fused Deposition Modeling (FDM), Selective Laser Sintering (SLS), and others in accordance with international standards like ISO and ASTM, as depicted in Figure 1 [2,8,10,11,24]. 

Fused Deposition Modeling (FDM) is commonly employed in medical and dental applications due to its cost-efficiency and versatility, yet it is hampered by limitations in printing resolutions, rendering it less suitable for intricate anatomical fabrications [7]. Typical materials in use encompass acrylonitrile butadiene styrene (ABS), polylactic acid (PLA), polycarbonate (PC), and polyamide (nylon) [1,4,7]. In contrast, Selective Laser Sintering/Melting (SLS/SLM) and Powder Bed Fusion (PBF) harness high-density lasers to produce dental implants, metal products, and ceramic restorations boasting superior printing quality when compared to FDM [1,4]. Nevertheless, these techniques come with elevated operational and material costs, as well as the potential for cooling-induced warpage, finding their niche in dental applications for crafting biocompatible dental implants and orthodontic appliances that not only reduce appointment frequency but also enhance patient comfort [1,7,21].

Another 3D printing technique utilizing photosensitive resin materials includes polyjet (PJ) and vat-photopolymerization (VP), which stand as favored choices for clear orthodontic aligners due to their capacity to cure and model materials under light irradiation [4,25]. The quality of prints, encompassing accuracy, durability, and aesthetics, hinges heavily on the viscosity of the resin used [26]. Polyjet (PJ) printing, employing UV-cured photosensitive resin and offering high resolution and material versatility, holds particular value in orthodontics for generating stage-specific models tailored for clear aligners [1,4,7,27]. Nevertheless, it comes with certain drawbacks, such as extensive post-processing, high material costs, and limited long-term durability [1,4,7].

Among the various techniques employed in dentistry, the UV-curable VP process has emerged as the most suitable option in dental applications, excelling in terms of accuracy and precision when compared to material jetting processes, offering superior product aesthetics and clarity compared to the fusion processes, and demonstrating greater affordability in comparison to the alternative UV-cure process, PJ. VP is a process that employs UV light to cure liquid photopolymer resins in a vat, building the object layer by layer. These processes use liquid photopolymer resins that consist of (meth)acrylate monomers, oligomers, and photo-initiators. The polymerization process occurs after photo-initiators generate free radicals upon exposure to a specific UV wavelength. The monomers and oligomers then start to form bonds via the chain radical polymerization mechanism [11,19]. The VP 3D printing process can be further classified based on the employed light source and pattern, using techniques such as stereolithography (SLA), liquid crystal display (LCD), and digital light processing (DLP) [28]. Compared to FDM and SM techniques, the VP method shows higher surface properties, precision, and accuracy [11,22]. 

SLA is the earliest practical 3D printing technology. The device consists of a reservoir for the material of photosensitive liquid resin, a build platform, and a UV laser light source [4]. SLA creates parts in a layer-by-layer sequence by photopolymerization. A UV laser is used to then spot-cure the liquid resin on the immersed building platform. After a single layer of resin is polymerized, the build platform then moves the specified layer height, ranging from 15–150 μm. This allows the remaining uncured resin to fill and cover the previously cured layer. This process then repeats until the entire object is constructed [1,4]. The advantages of SLA include high-temperature resistance and printing complicated geometric figures [1]. In dentistry applications, SLA is generally used in designing individual surgical guides, implants, and producing temporary crown bridges [1,4]. However, an application in orthodontics via SLA has yet to be reported. 

DLP is the other form of VP that utilizes a microsystem consisting of a rectangular arrangement of mirrors, referred to as a “digital micro reflector”. Each mirror represents a singular pixel, and the overall printing resolution of a projected image depends on the total number of mirrors. Because the angles of each micro-reflectors can be adjusted individually and freely, the light emitted from the light source is then refracted by these micro-mirrors and is projected onto the surface of the liquid resin to initiate polymerization [4]. A major concern of DLP is the existing interlayer lines on the surface of the printed part when compared to those of SLA, which are less visible. Due to the combination of high accuracy and short printing duration of DLP, this printing method is considered to be suitable for dentistry and orthodontic applications [22]. Specifically, DLP is found to be the most suitable option in the fabrication of clear aligners from clear resins [10]. 

Several studies have provided comparative assessments between 3D printing techniques (see Table 1) and conventional dental manufacturing methods [19,29,30]. For instance, Simoneti et al. [29] evaluated surface roughness and mechanical attributes such as microhardness, flexural strength, and biofilm formation in samples created via SLA and SLS methods. The SLS samples demonstrated high surface roughness and commendable mechanical properties, whereas SLA samples exhibited satisfactory surface roughness but were lacking in mechanical strength. Park et al. [30] focused on the flexural strength of dental prostheses, comparing three 3D-printing technologies to traditional methods. They found that prostheses fabricated using DLP and SLA technologies with polymethyl methacrylate (PMMA) showed superior flexural strength compared to those made with traditional self-curing PMMA. However, FDM-generated samples exhibited dents, indicating lower strength. Venezia et al. [19] analyzed the precision of orthodontic models with crowded and aligned teeth produced via DLP, LCD, and SLA. While there were accuracy differences across the technologies, all were clinically acceptable. SLA printers, due to their point-by-point laser beam methodology, yielded better definition in complex areas but were potentially the least repeatable. DLP technology has an additional advantage of reduced printing time, as it polymerizes an entire layer in a single laser irradiation through pixel projection [4,11,28]. However, the SLA method may have slightly higher deviations in specific components than DLP [22]. The printer choice solely depends on the workflow suggested by the manufacturers since following the proper steps of manufacturing would lead to proper safety and performance of the 3D printed parts. While considering the chairside treatment, the workflow time becomes an important factor and thereby the DLP-based workflows may be preferred [4]. 

## 5. Applications of 3D Printing in Dentistry 

Digital dentistry and 3D printing have rapidly gained prominence in the field of dentistry, offering several advantages such as rapid production, high precision, and reduced patient discomfort [1]. The integration of intraoral scanning technology has further expanded the utility of 3D-printed dental models for personalized patient care [22]. Ongoing research efforts are actively exploring 3D printing’s potential in dentistry, with a focus on enhancing production efficiency, quality, and treatment timelines. The dental implants and dentures sector is poised for substantial growth, driven by an aging population and the increasing demand for cosmetic dentistry. Projections indicate that the global dental 3D printing market is set to reach $3.4 billion by 2025, a significant rise from $903 million in 2016 [1].

**Figure 2:**
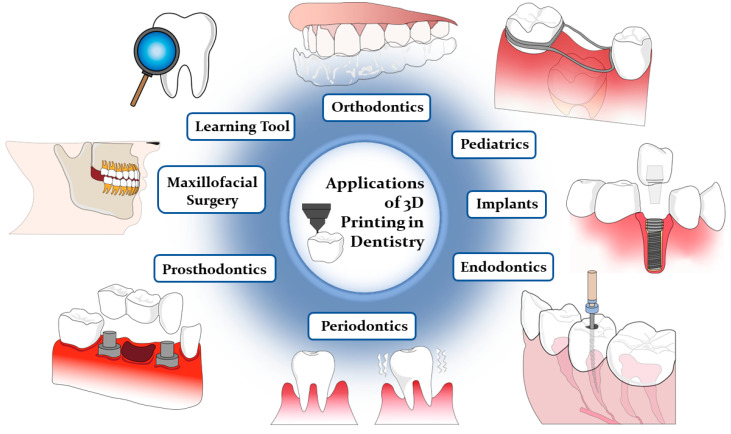
Applications of 3D printing in dentistry. Adapted from [3,6,7].

The transformative impact of 3D printing on dentistry is evident through its integration of CAD/CAM technology, oral scanning, design, and additive manufacturing [1]. This technology facilitates the rapid and direct creation of anatomical models to support a broad spectrum of dental applications [22]. In a general context, 3D-printed models prove to be highly effective as training aids, significantly reducing fabrication, sterilization, and post-production times, thus providing valuable support for diagnostic and procedural purposes [6]. More specifically, these models find applications across various dental specialties, including prosthodontics, oral and maxillofacial surgery, oral implantology, endodontics, periodontology, orthopedic implants, and orthodontics, enabling the production of medical devices such as aligners, retainers, veneers, and implants (see Figure 2) [2,4,5,10,23,24,33,35,36]. Across all of these aforementioned specialties, 3D printing has demonstrated its ability to significantly enhance the accuracy, precision, and patient comfort with surgeries and restorative dental procedures, benefiting both pediatric and general dentistry alike [1,2,3,4,6,7,8,37]. Delving further into the realm of specialized dental applications, it is important to recognize the ubiquity and significance of orthodontics. Simplifying treatments to maximize patient comfort and effectiveness while reducing fabrication time has been a driving force in this field, and 3D printing continues to play a pivotal role in achieving these goals.

Orthodontic practice fundamentally addresses malpositioned teeth and jaws, as well as malocclusions, misalignments between the dental arches [6,38]. Treatment often adheres to Sandstedt’s pressure-tension theory, where an externally applied force compresses the periodontal ligament (PDL) between the tooth and alveolar bone, facilitating bone formation and subsequent tooth realignment [38]. Traditional orthodontic approaches primarily utilize gypsum or plaster models of dental arches, which are cumbersome, fragile, and subject to error [4,6]. Therefore, the treatment often incorporates 3D-printing technology to correct malposed teeth through external force application [6,38]. However, conventional steel-wire braces are aesthetically challenging and uncomfortable, whereas clear aligners offer improved aesthetics at the cost of more frequent orthodontic visits [3,38]. 

The integration of 3D printing and digital scanning technologies has revolutionized orthodontic practice [3,6]. Utilizing oral scanners and orthodontic design software, e.g., 3Shape, accurate dental arch scans are crucial for malocclusion identification and treatment planning [4,6]. 3D printing allows personalized orthodontic treatment, including angulation, bending, and material selection for brackets or aligners [2,7]. Customized orthodontic solutions, such as patient-specific brackets and night guards, are now feasible through the synergy of intraoral scanning and simulation software [21,39,40]. For instance, 3D-printed polycrystalline alumina ceramic brackets, used in conjunction with an indirectly bonded tray, facilitate efficient tooth alignment [21]. 3D printing surpasses traditional methods in fabricating aligners by providing rapid, accurate, and efficient outcomes [4,6,41]. Moreover, these printed technologies offer advantages in terms of material durability, data digitization, and environmental sustainability [4,6]. 

As 3D printing gains widespread recognition, it has given rise to the development of 3D printable resins, serving as a versatile alternative to dental stone and enabling the fabrication of transparent teeth alignment systems [6,21,42]. These systems are typically crafted from a range of common polymers, including polyester (PE), polyurethane (TPU), polypropylene (PP), polyethylene terephthalate (PET), polyethylene terephthalate glycol (PETG), polycarbonate (PC), ethylene vinyl acetate (EVA), and polyvinyl chloride (PVC) [25,27,36,43]. The choice of these materials is rooted in their mechanical, optical, and physical attributes [25]. For instance, TPU boasts numerous advantageous properties, such as chemical resistance, abrasion resistance, adhesion characteristics, and ease of processing. However, it should be noted that TPU is not inert and is susceptible to the effects of heat, moisture, and prolonged contact with salivary enzymes. Furthermore, PC shares many similarities with polymethyl methacrylate (PMMA) but surpasses it in terms of superior mechanical and optical properties, while also exhibiting effective functionality across a broader temperature range. Another common material, PETG, is renowned for its excellent creep properties, fatigue resistance, remarkable flexibility, and formability but has limited chemical resistance to the typical solvents employed in dental 3D printing [26,27].

In the context of aligner design, an optimal configuration involves the incorporation of both hard and soft layers, where the hard layers contribute to robustness and durability, while the soft layers prioritize wearer comfort [36]. Research findings have indicated that the efficacy of aligner therapies is notably enhanced when a multi-stage treatment approach is employed, complemented by the inclusion of auxiliary elements to augment force delivery. Furthermore, a growing body of evidence underscores the versatility and effectiveness of clear aligners in addressing a wide spectrum of orthodontic challenges. These challenges encompass issues such as anterior crowding, deep bites, arch expansion, buccolingual tipping, crown movement, molar distalization, teeth rotation, extrusive tooth movement, crossbites, anterior teeth intrusion, anterior open bite, overbite reduction, class-II and class-III malocclusions, rotation-uprighting of bilateral winged maxillary central incisors, space closure, and various other complex cases [27,43,44,45,46,47]. For a comprehensive assessment of clinical treatment effectiveness and efficiency across various cases of tooth movement, readers are directed to a comprehensive comparative study conducted by Yassir et al., which offers valuable insights into the comparative outcomes of clear aligners versus fixed appliances [32].

Even though clear aligners offer advantages in simplicity, comfort, and removability, preserving esthetics and supporting oral hygiene, they still face several challenges, including treatment time, accuracy, attachability, cost, accessibility, and biocompatibility [25,34,44,48,49]. Treatment duration depends on patient compliance, and challenges can arise with lower premolars having a round morphology [48]. Clinical studies have revealed varying levels of treatment accuracy, indicating the need for further research [50]. Prediction models have shown approximately 78% accuracy in aligner therapy outcomes [27,51]. Some thermoplastic polymers used in aligners can exhibit cytotoxic effects due to monomer release during thermoforming [43,48]. Additionally, conventional aligner manufacturing limits appliance changes during treatment [48]. Researchers have observed material degradation related to temperature changes and water absorption, influenced by polymer crystal structure [2,10,42,52,53]. Temperature and long-term intra-oral use can affect surface morphology and mechanical properties [9].

## 6. Aligners

**Figure 3:**
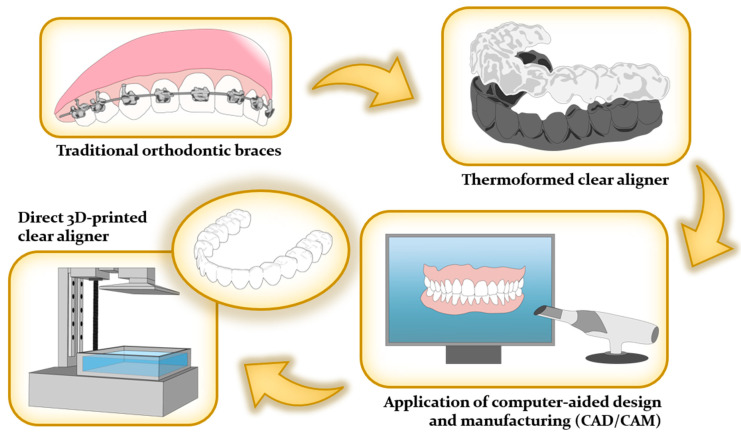
Evolution of orthodontic appliances in dentistry.

The historical evolution of aligner technology can be traced back to Dr. Harold D. Kesling’s groundbreaking work in 1945, where he pioneered the use of plastic-based tooth aligners for addressing minor malocclusions. Kesling not only laid the foundation for this approach but also provided insights into its limitations and future potential [54,55,56]. Building upon Kesling’s work, Henry Nahoum made significant contributions in 1964 by introducing vacuum-formed thermoplastics and auxiliary components, enhancing the precision and effectiveness of corrective forces [31,36,47,57]. In 1971, Robert Ponitz emphasized the use of transparent materials and incremental staged movements, further advancing orthodontic alignment techniques [58]. A pivotal moment in modernizing aligner technology occurred in 1997 when Zia Chishti and Kelsey Wirth introduced the CAD/CAM-based Invisalign™ system, which has since undergone multiple iterations to optimize its efficacy [58,59,60,61]. Presently, the integration of 3D printing technologies in aligner fabrication has sparked a revolution in customization and production efficiency, as demonstrated in Figure 3.

To address these challenges, a cutting-edge solution of the direct 3D printing aligner has emerged. Its primary objective is to minimize reliance on external processing by seamlessly integrating 3D scanners, software, and printers, granting orthodontists complete control over the workflow. This effectively eliminates the disadvantages associated with outsourcing, including cost and lead time constraints [55,62]. Notably, in-house clear aligners prove highly advantageous for uncomplicated cases, enabling cost-effective and expeditious treatment attainment [55,63,64]. Moreover, the incorporation of Computer-Aided Design/Computer-Aided Manufacturing (CAD/CAM) techniques in in-house clear aligner treatments has significantly enhanced production efficiency. It empowers orthodontists to closely monitor and adjust tooth movements at each treatment stage [55,64]. Noteworthy software innovations, like Bio-CAD, streamline these processes, minimizing the need for manual intervention [65]. These software programs also excel in tracking 3D forces required for precise tooth movements, a pivotal aspect of orthodontic care [1]. Furthermore, advancements such as preoperative planning, digital techniques for fixation and positioning during surgery, and presurgical orthodontic support have created a seamless transition for orthognathic cases. This expanded capability enables 3D printing clear aligners to address even more intricate scenarios [66]. Importantly, the production of clear aligners can be repeated without limitations [64].

Moreover, the direct 3D printing of clear aligners can mitigate issues caused by conventional thermoforming. This technological evolution addresses concerns regarding alterations in material properties [19,25,27,60]. For instance, digital modeling in dentistry has led to clear, removable splints for orthodontic restorations [25,37]. 3D printing is transforming dentistry, enabling precise, customized, and reproducible solutions [7,67]. A study conducted by Thurzo et al. [17] showed that the utilization of a 3D-printed orthodontic distalizer in treating a complex case, such as class-II unilateral malocclusions. while pleasing the patients due to its transparency.

Furthermore, 3D-printed dental resins exhibit remarkable color stability compared to conventional counterparts, particularly during extended post-curing periods [11,68]. These dental resins possess a range of essential characteristics that are considered ideal for orthodontic materials, including substantial elastic retention, high toughness, excellent formability, low stiffness, biocompatibility, and environmental stability [27].

In addition to enhanced color stability, 3D printing offers the advantage of customizable intra-aligner thickness, reducing the necessity for attachments, albeit at the potential cost of reduced transparency due to current material choices [10,42]. The thickness of the printed aligners can be tailored within a range of 0.25–1.2 mm, utilizing materials resistant to dimensional deformation during the manufacturing process [42,69,70]. Notably, the relative affordability of 3D printers in recent times has made them more accessible in dental and orthodontic offices. This surge in availability has led to increased popularity in the use of in-office aligners, subsequently reducing lead times and costs for patients [2,7,21,60]. Furthermore, ongoing advancements in technology and techniques are actively working to minimize potential material waste associated with non-recyclable thermosetting resins. This is achieved through the development of base models and orientations that demand fewer attachments or support during the printing process [21,71,72].

Finally, it is worth noting that 3D-printed aligners offer greater geometric precision tailored to specific customer requirements [41]. Maintaining uninterrupted contact between the aligner and the teeth is vital for ensuring its optimal performance, as any gaps or spaces can hinder the aligner from fully realizing its mechanical potential [62,71,73]. Additionally, 3D-printed aligners uphold robust mechanical properties, including hardness, indentation modulus, and elastic index, with only minor property degradation observed after a week of use. These aligners also effectively apply the necessary forces to achieve the desired tooth movements [71].

Several FDA-approved materials are offered for direct printing of 3D orthodontic aligners. One prominent example is the product suite developed by LuxCreo, which has obtained FDA Class II 510(k) clearance for directly 3D printed clear dental aligners. This suite includes a range of digital fabrication tools and materials specifically designed for creating dental aligners [74]. Additionally, other commercially available resins for producing clear aligners include Dental LT, TC-85 (Graphy, Seoul, Republic of Korea) [36,41,71,75]. The characteristics of these aligners are influenced by a variety of printing methods, encompassing factors like cellular toxicity, force application, flexibility, and viscoelastic properties [41,75,76]. Note that the published properties of the two commercially available materials are different from that of thermoplastic aligner properties, thereby the clinical efficacy must be carefully examined.

These developments represent a growing trend in the dental industry towards adopting 3D printing technologies for more efficient and accurate production of dental appliances, including aligners. The FDA approval of these materials is a crucial step in ensuring their safety and efficacy for patient use.

## 7. Required Properties of the 3D Printed Aligners

**Figure 4:**
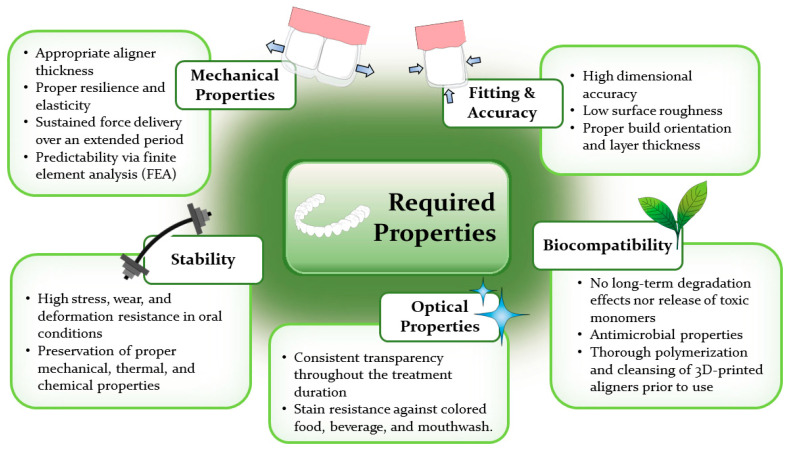
Required properties of 3D-printed clear aligners.

The successful formulation of treatment plans involving clear aligner therapy necessitates a thorough evaluation of the diagnosis and the thoughtful selection of key aligner features, such as their mechanical properties, thickness, and activation level [42]. To ensure the quality and safety of 3D-printed dental resins used in orthodontic devices, the necessary specifications for physical and mechanical properties are outlined in ISO 20795-2 [76,77]. Apart from the mechanical characteristics, other crucial aspects that demand careful consideration include fitting precision, accuracy, stability in intraoral environments, optical appearance, and biocompatibility of the 3D-printed aligners, as concisely summarized in Figure 4.

### 7.1. Mechanical Properties

One of the major problems found in current thermoformed clear aligners is that they need refinement, midcourse correction, adjunct fixed appliances, and sometimes even retreatment with fixed appliances [47]. In order to construct proper clear aligners via 3D printing, parameters such as material selection, aligner’s thickness, force delivery, stress relaxation, and prediction should be thoroughly considered. 

#### 7.1.1. Elasticity and Force Delivery

In planning an orthodontic treatment with an aligner system, it is important to perform a biomechanical analysis of the printed material and appliance to know the exact distribution of the forces and moments [78]. Compared to conventional fixed appliances, clear aligners cover the entire dentition as an overlay appliance during teeth movement, making them significantly different in effectiveness and accuracy [79]. Aligner-based orthodontic treatment involves incremental movement of teeth by multiple successive aligners or trays; each of which progressively repositions teeth by small amounts [47]. 

##### Thickness

The mechanical properties of aligners are intricately linked to their thickness [75,80]. Variations in thickness significantly affect force-deformation properties, with thinner aligners exhibiting reduced stiffness and diminished force transmission [81]. Notably, 3D-printed aligners gain strength as layer thickness decreases, highlighting the role of thickness in force generation [4]. Aligner thickness directly influences the force magnitude, with thicker appliances delivering notably higher forces compared to their thinner counterparts [42,82]. Aligners with increased thickness exhibit elevated values of modulus of elasticity and lower deformability under load, making them more suitable for translation or root movement [47,75].

Reducing thickness amplifies bodily aligner deformation, consequently reducing tooth-to-aligner contact areas. This decrease in thickness also enhances flexibility but comes with an increased risk of fracture [81]. Thinner materials are better suited for producing lighter forces suitable for tipping, while translation or root movement necessitates thicker aligner material [47]. Consequently, careful consideration of aligner thickness is essential to establish an appropriate mechanical environment for optimal tooth movement over time [42].

Conversely, low thickness results in a low modulus of elasticity and high deformability of aligners [75]. Aligner thickness generally falls within the range of 0.5 mm to 1.5 mm, as indicated by literature and various manufacturers [42,47]. Thermoformed aligners typically span 400 to 1500 μm, leading to the selection of three common thicknesses: 250 μm, 500 μm, and 750 μm [80]. For instance, 0.75-mm transparent aligners achieved 95% lingual inclination rotation and 76% axial rotation, while 0.5-mm transparent aligners induced 79% lingual inclination rotation and 70% axial rotation, relative to fixed appliances. The increased effectiveness of the 0.75-mm aligners likely stems from their greater force application [38].

During the treatment course, aligner thickness and material properties should be adjusted as needed [81]. A new aligner design protocol suggests a uniform thickness of 0.5 mm for one week and 0.7 mm for ten days of wear [62]. Recognizing that irregular aligner thickness can complicate treatment outcomes, the use of 3D-printed aligners is proposed to achieve uniform thickness across the entire surface [80,81]. However, factors contributing to thickness deviation may still arise, such as residual resin incorporation and differences in resin types, including shrinkage, light reflection during scanning, and spray interaction, all potentially affecting thickness and 3D surface deviations. These thickness deviations can have clinical significance, impacting aligner seating, force application, and the available area within the aligner for tooth movement [42].

##### Resilience and Elasticity

The material thickness is not the only factor that affects the retention of aligners [83]. An ideal aligner should display resilience and elasticity while maintaining its properties statically through treatment [9,25]. An aligner material should ideally possess adequate stiffness to exert the forces and moments needed to achieve the planned tooth movement while allowing the aligner to hold firmly to the teeth with high retention force [36,83]. From a mechanical standpoint of view, the decrease in modulus implies attenuation of the force delivery capacity by the appliance during intraoral use [9]. In the case of tooth tipping, a high degree of aligner’s elasticity is required to rebound and straighten the tooth. Moreover, an adequate modulus must be high enough to be sustained in the mouth for a period of time to allow the tooth to move per the treatment plan [84]. One way to increase the stiffness is to have a straight extended design. This leads to a better stress distribution and better control of tooth movement but also applies more force at the gingival area, closer to the center of resistance, which can potentially improve control of bodily movement [81]. However, if an aligner possesses excessive stiffness, patients may have difficulty during placement and removal while an aligner with low stiffness cannot generate adequate forces required to move teeth [36].

The flexural modulus serves as a fundamental parameter that characterizes how a material responds to stress and strain when subjected to flexural deformation. It provides insight into a material’s ability to withstand bending forces [78]. Presently, the established standards for orthodontic base polymers adhere to criteria outlined in JIS T6528 [85] and ISO 20795-2, mandating minimum values of 50 MPa for flexural strength and 1300 MPa for flexural modulus [76]. Nevertheless, it’s important to note that there are currently no specific standards in place for orthodontic aligners.

An ideal orthodontic aligner is expected to exhibit rigidity, possess a high yield strength, and maintain the ability to deliver forces within an elastic range. However, typical materials used for aligners exhibit an elastic modulus approximately 40 to 50 times lower than that of conventional Ni-Ti archwire. This substantial difference in modulus indicates that aligner materials are significantly more prone to permanent deformation when compared to most archwire appliances [47].

##### Force Delivery

To facilitate effective tooth realignment with aligners, maintaining appropriate stress levels throughout treatment is essential [47]. These exerted forces and their precise delivery depend on factors such as point of application, magnitude, direction, and the Center of Rotation (CoR) of the tooth [81], all while staying within safe limits to prevent tooth damage [72]. The quality of orthodontic force from clear appliances hinges on fabrication material properties [27], and digital setups enable precise execution of individual tooth movements. This precision is crucial given its relevance to aligner therapy forces [18]. Tooth movement involves an interplay of stress between the appliance and the biological complex of the Periodontal Ligament and surrounding bone [47]. In biomechanics, there are two types of tooth movement: tipping, where the crown moves while the root tip stays stationary, and bodily, where both crown and root move simultaneously. The type of movement depends on the applied force’s relation to the CoR and its location on the tooth. Achieving pure bodily movement necessitates that the force passes directly through the CoR, typically situated one-third of the root length apical to the alveolar crest for single-rooted teeth [58]. However, this location varies based on surrounding bone, root length, and shape. Ideally, directing force at the CoR produces orthodontically preferred bodily movement. Challenges arise when roots are deeply embedded, necessitating gentle application of continuous forces to the accessible crown for effective realignment [86].

There are two primary methods for applying forces to a tooth. The first involves applying a single force directed away from the CoR, referred to as the “moment of force”. This force causes the CoR to shift along the force’s line of action, resulting in the tilting of the tooth around the CoR. The second method entails applying a pair of equal forces, forming a “force couple,” which generates a rotational tendency, commonly known as a “moment of the couple” [58].

In the aligner system, applying biomechanical techniques like fixed braces is challenging due to varying forces from incisal to gingival regions and a geometrical mismatch between tooth and aligner. Predictability of bodily movement in clear aligner treatment is limited as stresses are dispersed over a broader contact area [81]. Aligners move teeth through pushing, with better intrusion capabilities than extrusion. Clear aligners primarily induce crown tipping, while root torquing is less predictable [47]. Achieving bodily translation requires balancing forces at the incisal edge and gingival crevice, avoiding tipping [81]. Controlled movement necessitates precise force titration and moment application [47].

However, the setup model’s deviations, influenced by factors like malocclusion and aligner properties, can result in variable forces affecting tooth movement [18]. Excessive forces can lead to side effects like root resorption and patient discomfort [18,73,78]. Intrusive forces are particularly concerning, as they commonly lead to root resorption during various tooth movements [87]. Lateral incisors are especially vulnerable due to force concentration on their smaller root surface area [88]. Inadequate forces hinder effective tooth movement [78], necessitating careful consideration of undesirable forces and moments during aligner-based orthodontic treatment planning [88]. To mitigate these issues, it is crucial to apply mild forces initially to protect teeth and surrounding tissues. The choice of aligner stiffness, gap volumes, and their positions significantly impacts treatment success. Foil thickness directly correlates with force delivery, with thinner foils being more flexible and suitable for the initial phase of aligner treatment. As treatment progresses, thicker foils can be used to apply higher orthodontic forces [87].

In axial rotation, transparent aligners can only transmit force to the crown region; this may cause transparent aligners to exert lower forces than fixed appliances. This suggests that transparent aligners do not transmit sufficient force for the treatment and that axial rotation correction using transparent aligners may require additional orthodontic treatment [38]. Traditionally, by using a wire or bracket, changing the type (modulus) of the archwire and dimensions (moment of inertia) can create the desired type of tooth movement to give varying intensities of a force system [47].

Optimal force delivery is crucial for effective orthodontic treatment, achieving maximum tooth movement rates without harming teeth. For various tooth movements, recommended force ranges are as follows: 0.5 to 0.75 N for tipping, 1 to 1.5 N for rotation, 0.75 to 1.25 N for torque control, and 0.75 to 1.25 N for bodily movement [78]. In clinical practice, orthodontic forces typically range from 0.098 to 1.18 N, depending on the specific type of tooth movement [73]. Aligners aim for tooth movement in the range of 0.2 to 0.3 mm for translations and 1 to 3 degrees for rotation over a 14-day period [61,89,90]. Recent studies have shown that orthodontic forces generated by aligners range from 0.18 to 2.91 N, similar to those delivered by Ni-Ti wires in traditional therapy [91]. Consequently, 3D printers must produce dental models with accuracy errors below this range to fabricate effective orthodontic aligners. It is worth noting that aligners fabricated using entry-level 3D printing systems may not achieve the intended orthodontic movements compared to those made with professional devices [19]. 

Evaluating the forces exerted by clear aligners on attachments is essential for comprehending tooth movements and achieving desired results with minimal tooth damage [72]. Two common methods for measuring force and pressure delivery involve using pressure-sensitive film to create a pressure distribution map of the tooth’s contact area or employing a strain gauge mounted on a specific tooth [72,81,92]. Research has revealed that these forces and stresses are often unevenly distributed across the entire facial tooth surface [81]. Such measurements provide valuable insights for optimizing treatment outcomes.

A well-fit, well-retained aligner is essential for transmitting higher forces and achieving accurate tooth movement. During upper central incisor movement with an orthodontic aligner, stress does not evenly distribute across the tooth surface; instead, it concentrates in specific force application areas. Additionally, aligner-generated forces vary with trimming design width and extension, with non-extended aligners producing notably lower forces than extended ones. Uneven tooth surface topography significantly influences stress distribution, resulting in uneven stress dispersion. When the aligner and tooth do not make full contact, areas of relief differ across the tooth surface [81].

#### 7.1.2. Resiliency

Resiliency refers to a material’s capacity to absorb energy while under elastic loading and release that energy upon unloading, all without causing permanent, plastic deformation [36,75]. Resiliency is quantifiable by assessing the area under the stress-strain curve up to the elastic limit. In comparison to archwires, aligners exhibit notably lower resiliency, as they absorb less energy before undergoing permanent deformation, especially when subjected to moderate-to-heavy loads [47]. It is important to note that the energy absorbed by aligners is predominantly dissipated as heat, with a relatively minor portion transferred to the teeth [36,47].

#### 7.1.3. Viscoelasticity

Viscoelasticity is a critical property of aligner materials, as it pertains to their ability to absorb shocks, vibrations, and forces [47]. It’s important to note that viscoelastic material properties can undergo significant changes over time, beginning from the moment force is applied, even before planned tooth movement begins [36].

#### 7.1.4. Stress Relaxation

Stress relaxation refers to the time-dependent decrease in stress under constant strain conditions. It gauges the consistency of force delivery over time and significantly impacts aligner efficiency [36,47]. Clear aligners, owing to their formability and viscoelastic nature, can exhibit varying behavior over time when subjected to loading [25,27]. This variation includes self-relaxation, where loads decrease over time while maintaining constant deflection [25]. It is characterized by applying a fixed deformation and tracking the load required to sustain it over time [47]. In orthodontics, maintaining a consistent force is desirable, but aligners often fall short in this regard [36,47].

Viscoelastic materials like aligners experience deflection increases over time when subjected to constant loads, and the decrease in force isn’t linear but rather follows an exponential trend [36,47]. There is a significant initial drop in force, indicating material fatigue, particularly within the first few hours of use, and a similar rapid drop when torque is applied. When planning aligner-based treatments, accounting for this force drop is essential [47]. 

Moreover, the stress relaxation process is influenced by factors such as the material composition of the aligner, oral cavity temperature, applied force magnitude, and material thickness. Monolayered materials exhibit greater resistance to stress and slower stress relaxation, while multi-layered materials demonstrate more consistent stress relaxation and lower absolute stress resistance [36]. These considerations are crucial in understanding and optimizing aligner performance.

In clinical situations, aligners are subjected to both short-term and long-term forces in the oral cavity. When an aligner is fitted onto the dentition, the aligner material is loaded with short-term forces after the immediate fit-in. When the aligner is worn for a significant duration, it experiences long-term forces due to the displacement caused by the planned tooth movement and reactionary forces generated by the musculoskeletal system [36].

#### 7.1.5. Toughness 

Toughness is a crucial property for clear orthodontic aligners, ensuring their durability, reliable force application, and patient comfort throughout treatment. Therefore, it is a property that needs to be optimized for improving the quality of orthodontic products. Several considerations demand attention:

- Resistance to fracture: Aligners face diverse challenges—biting forces, repeated handling, and occasional mishaps—without succumbing to cracks or fractures. Ma et al. [93] suggest a minimum flexural strength of 50 MPa and a fracture toughness exceeding 1 MPa·m^1/2^ as benchmarks for adequate resilience.
- Resistance to deformation: Maintaining predictable shape and consistent force delivery throughout treatment is crucial. Duran et al. [94] propose a modulus of elasticity between 1500 and 2500 MPa, striking a balance between effectiveness and patient comfort.
- Resistance to fatigue: The repetitive insertion and removal cycles can induce fatigue cracking. Gold et al. [95] emphasize the need for materials with intrinsic fatigue resistance to prevent premature failure and ensure extended aligner performance.
- Resistance to wear: Friction and abrasion within the oral environment can compromise aligner fit and efficacy. Weir [96] underlines the importance of materials with low wear rates and smooth surfaces for optimal long-term function.

Addressing these toughness requirements necessitates continuous material development and advancements in fabrication processes. Aligner manufacturers diligently explore innovative solutions to enhance strength, flexibility, and patient comfort, constantly pushing the boundaries of what these clear, transformative tools can achieve.

From the material science standpoint, toughness as a material property involves increasing the amount of energy absorbed before the failure [97,98]. There are a few techniques to increase toughness. One effective method to increase toughness is to increase the molecular weight of the polymer chains [99]. It can be achieved by either selecting the oligomers with a higher molecular weight or through curing and posturing. For instance, the extend of polymerization can be increased via post-processing techniques, such as utilizing a UV-heat-dual-component curing system [100]. Another method to increase toughness includes utilizing multi-component systems by introducing different printing materials (i.e., polymers, metals, and ceramics) or nanofillers to increase toughness [101,102,103]. It should be noted that even though utilizing higher molecular weight oligomers can enhance toughness of the aligners, it can also increase the overall viscosity of the printing resins, which affects processibility. Therefore, the processing limitations need to be considered during the materials selection to ensure the successful fabrication of a durable medical device [102].

#### 7.1.6. Application of Finite Element Analysis and Prediction

Tooth movement plays a pivotal role in the success of orthodontic treatments. Transparent aligners must effectively transmit orthodontic forces while avoiding harm [38]. Biomechanical experiments and finite element analysis (FEA) are applied as essential tools for evaluating these forces and tooth mobility, enhancing the predictability of clear aligner therapy [38,44]. FEA, which is a 3D simulation technique, replicates real-world conditions to predict biomechanical effects [38,104] and facilitates the simulation of orthodontic treatments, including the study of complex phenomena [38]. In medical device development, FEA is invaluable for data comparison and creating virtual environments. It becomes indispensable for optimizing attachment shapes and positions in aligner treatments, especially when dealing with four distinct tooth movements, each requiring specific attachment positioning [104].

FEA is known for its efficiency in studying dental biomechanics and orthopedics [38]. It allows for comprehensive assessments encompassing material suitability, mechanical behavior, and manufacturing processes [26,105]. In clear aligners, tooth displacement arises from intentional tooth-aligner mismatches, generating significant stress within the aligner itself, despite the small discrepancy (approximately 0.25 mm and less than 3° for rotation). FEA has revealed that high aligner activation, especially for tooth rotation with attachments, can result in stresses up to 3.7 MPa and deformations of up to 300 microns [91].

Several studies have explored the performance of clear dental aligners under compressive mechanical loading equivalent to human bite force using FEA. They found that these aligners can withstand non-linear, cyclic compressive forces akin to human biting forces while boasting qualities such as geometric accuracy, rapid production, biocompatibility, 3D printability, and mechanical strength [105]. Notably, a biomechanical finite element study determined that a force as low as 0.1 N is sufficient to initiate orthodontic tooth movement without inducing hydrostatic stress in the canine periodontal ligament (PDL) [88].

However, no study that conducted FEA reflected the actual orthodontic treatment environment [38]. None of the aligner systems take into consideration the anatomy of the root, specifically the location of the CoR nor the prediction for the force system, specifically the moment of a force [47]. In orthodontics, the treatment method using aligners cannot deliver an adequate load for effective and stable movement of teeth [104]. 

The combination between biomechanical assessment, analysis of aligners and attachments using the FEA can lead to a more effective movement of teeth [104]. FEA results thus provide a significant guideline for the mechanical load-bearing capacity of these aligners, which can further motivate scientists and dentists to conduct other mechanical load tests experimentally [105].

### 7.2. Fitting and Accuracy

The exact fit and tolerances of clear aligners are indispensable for successful treatment since they must seamlessly adapt to the teeth; conversely, overbuilding the aligners can impede proper seating and diminish the effectiveness of orthodontic forces. To ensure the correct aligner thickness, 3D surface deviation maps are employed, revealing potential deviations from tooth contact, which may result from factors in the 3D printing process, initial scanning, superposition errors, post-processing conditions, or a combination of these elements [42]. The accuracy of the model used to create clear aligners directly impacts the precision of subsequent tooth movements. In this regard, intraoral scans represent the most accurate method for aligner fabrication and fit [61]. Additionally, the resolution of digital scanning systems can influence overall accuracy [19]. Enhanced dimensional precision in the z-direction can be achieved by integrating light absorbers in the 3D printer to control the curing depth [11].

Hence, the development of 3D scanners, materials, and printing devices assumes a crucial role in attaining aligners with superior dimensional precision, refined surface quality, enhanced aesthetics, and heightened clinical utility [11]. Furthermore, variables like arch size (complete-arch or quadrant-arch), 3D-printing techniques, and measurement methods can exert an influence on the accuracy of dental printed components [22]. Research conducted on economical, open-source printers—originally not tailored for dental applications but evaluated against existing standards (ISO 5725-1 [106] and ISO 12836 [107])—has showcased remarkable accuracy, particularly in SLA printing [11,91]. Conversely, closed-source printers, purposefully designed with resin material properties and conversion rates in mind, offer a guaranteed and higher level of accuracy and surface quality for dental parts [11]. Therefore, when examining research results, it is crucial to consider whether the utilized printer in the study is of a professional or economic nature.

To achieve the desired tooth movement, alignment between the file and the model must be within 250 microns [18]. However, since aligners facilitate minor tooth adjustments, the deviation between the tooth and the aligner’s intended position should not exceed 120 microns [22]. Thermoforming typically results in a gap of 100 to 350 microns between the aligner and the tooth surface [18]. Maintaining high accuracy is crucial for a perfect fit and to prevent even minor misalignments during intraoral scanning. Such misfits can lead to biological issues and disrupt precise tooth movements, a core aspect of aligner treatment, potentially leading to undesirable outcomes. Clinical discrepancies exceeding 120 μm in width are considered unacceptable [22].

In aligner therapy, precision in force transmission relies on the seamless aligner-tooth fit. The accuracy of setup models is pivotal in achieving this. When using 3D-printed aligners, a deviation from the virtual model of over 0.25 mm must be avoided for precision. Recognizing and addressing manufacturing variations is crucial during setup, requiring adjustments in movement sequencing, especially in specific regions [18]. Fortunately, 3D printing provides a practical solution for these adaptations.

While DLP 3D printing is favored in the dental industry for its high precision, achieving optimal dimensional accuracy depends on factors like materials, build orientation, and layer thickness [22]. Aligner designs have a shell-like structure, with an inner surface (intaglio) contacting teeth and an outer surface (cameo) touching soft tissue and dentition. Complex designs should be carefully considered, as deviations from the digital model during printing can lead to clinical issues, including undesirable tooth movement [89]. Printing-related deviations often result from shrinkage and incomplete polymerization in resin monomers exposed to light sources [22]. These changes in resin 3D-printed structures can be attributed to factors like free monomers, layer spacing, and microstructural defects induced during printing (from UV/laser light). Over time, these post-printing dimensional shifts may cause issues such as dissolution, disintegration, delamination, and part swelling [90]. To mitigate this, post-polymerization is employed, though some shrinkage may occur [4,91]. While supporting structures enhance stability during printing, they should be removed after printing to preserve the aligner’s shape [71].

The part’s orientation during printing significantly impacts workflow efficiency and the mechanical characteristics of printed specimens, necessitating an assessment of its effects on both mechanics and dimensions [80]. Beyond its influence on object properties, orientation dictates how many parts can be accommodated on the printing platform. When nearing the platform’s boundaries, additional layers are needed for secure attachment, prolonging printing time and raising the risk of failure [89]. Furthermore, an increase in the number of layers correlates with heightened surface roughness, emphasizing the preference for the orientation that minimizes layer requirements [28,71].

It is important to note that printing properties are highly dependent on the resin used, and the same has been found regarding print orientation. Research found that using specific resin systems did not significantly affect the accuracy of aligners or only affected localized areas [75]. In general, vertical aligner positioning will decrease the print time because it allows for more prints per platform, however horizontal positioning allows for faster print jobs because fewer layers are used [71]. In addition, horizontal positioning requires fewer support structures [62]. It is important to note that the thickness and orientation of the support structure also impacts the accuracy of printed aligners, however the distribution and placement of the supports is significantly more influential than their thickness [11]. There is conflicting research on which positioning orientation is optimal, however because of the variety of resin systems available, it is difficult to create a “one-size-fits-all” solution [4,11,19,62,71,75]. 

Apart from ensuring geometric precision, print orientation also influences the amount of resin consumed during the 3D printing process. The most suitable printing orientation for the 3D-printed aligner remains a subject of debate. Nevertheless, various studies have demonstrated that employing 0°- and 90°-orientations results in the product exhibiting minimal dimensional variation [80,89,90,108]. One printing effect to be aware of when considering the shape geometry and print orientation is the cupping effect. Cupping occurs when a concave surface is facing the print platform and the surface tension of the resin causes an unequal distribution, affecting the print accuracy [80]. This cupping can trap air and cause voids to form [89]. 

### 7.3. Stability in Clinical Applications

The success of clear aligner therapy heavily relies on patients consistently wearing their aligners for approximately 22 h a day or a total of about 150 h per week. To prevent potential issues, the design process must consider the mechanical properties, including stiffness, hardness, and elasticity, to ensure they remain stable despite intraoral conditions and regular usage [87,91]. Factors such as temperature, humidity, and salivary enzymes can impact both the aligner and its mechanical characteristics [91]. An ideal aligner should evenly apply force over a designated timeframe to maintain control and prevent potential aligner failure and irreversible damage.

#### 7.3.1. Mechanical Properties

Throughout orthodontic treatment, teeth are subjected to various forces to guide them into their correct positions. Hence, it is crucial for aligner materials to withstand these stresses effectively [91]. Any alterations in the material properties of an aligner post-application can result in a loss of control over teeth movement during its use [91,109]. Additionally, an aligner must maintain its ability to exert forces consistently, even after repeated insertions and removals for meals and oral hygiene routines [25,87].

Aligners endure both continuous and intermittent forces from normal oral functions, including speaking, chewing, swallowing, teeth clenching, and grinding, reaching magnitudes of up to 500 N [36,110]. Cyclic forces, simulating chewing and swallowing, alter mechanical properties, leading to reduced wear resistance, increased brittleness, stiffness, and deformation [87]. This reduced wear resistance indicates lower hardness, making weaker aligner materials prone to attrition under occlusal stresses. Furthermore, persistent loading from opposing teeth diminishes exerted forces in intraorally aged materials. Lastly, temperature and water absorption changes can reduce material stiffness, resulting in decreased orthodontic forces. Therefore, preserving stability in the chemical and mechanical properties of materials in the oral cavity, even after exposure to moisture, body temperatures, and occlusal forces, is vital for achieving desired outcomes [9].

A study conducted by Can et al. [82] investigated the impact of in-vivo aging on mechanical properties using TC-85 resin for clear aligners, focusing on hardness, indentation modulus, elastic index, and indentation relaxation. They found that clear aligners are susceptible to degradation from factors like water, microbes, and fungi. Differences in structure between 3D-printed and thermoformed aligners may explain variations in mechanical properties. Thermoformed aligners benefit from aromatic groups, enhancing hardness in a similar indentation modulus range. Notably, 3D-printed aligners exhibited a significantly higher relaxation index compared to thermoformed aligners, indicating a greater susceptibility to secondary reactions, including hydrolytic degradation involving the ester moiety.

Numerous research studies have explored the optimal timing for placing 3D-printed aligners, with a focus on evaluating their tensile, compressive, and flexural properties. These properties can change due to ongoing polymerization after the initial curing process [89,110]. For example, tensile properties were observed to evolve over 1, 3, 5, and 7 days, displaying increased ultimate tensile strength and failure stresses alongside reduced elongation over time. Compressive testing revealed that compressive yield strength and ultimate compressive strength peaked at 5 days [91]. Additionally, post-cured aligners exhibited nearly 75% greater strength in compressive testing compared to their uncured counterparts [110]. Finally, flexural testing demonstrated the highest values for flexural strength and failure stress at the 7-day mark [91].

In additive manufacturing, once a part completes the printing process, it is in a state known as the Green State. In this state, the parts assume their desired shapes but have not undergone full polymerization [89]. To enhance their properties, such as stability and mechanical strength, additional exposure to heat and UV light is employed after printing [89,91]. Notably, post-print UV curing has demonstrated the ability to significantly bolster the rigidity and compressive mechanical strength of transparent aligners produced directly through 3D printing. It is important to emphasize that post-print processing steps are indispensable for ensuring the mechanical stability of these aligners [89]. Interestingly, aligners subjected to post-curing exhibit substantially higher resistance to compressive forces compared to their uncured counterparts, with higher post-curing temperatures proving more advantageous [75]. It is worth noting that the duration of the curing process, known as cure time, plays a pivotal role, as extended cure times are associated with increased polymerization [111]. However, it is important to recognize that this increase in polymerization does not occur uniformly throughout the cross-section of a sample [91]. The role of UV curing is particularly critical in polymerizing the outer shell of the aligners to eliminate any residual monomers on the surface, ensuring their quality and safety [62].

#### 7.3.2. Thermal Properties

Temperature can have a significant impact on the mechanical characteristics of polymers, particularly when their glass transition temperature aligns with ambient room conditions [9]. Exposure to humid environments induces a notable deterioration in the mechanical properties and glass transition temperature of photocurable resins over time [4]. To counteract this undesirable effect, it becomes crucial to carefully consider the glass transition temperature when selecting aligner materials, as a failure to do so might lead to an originally rigid structure adopting a rubbery consistency [36].

#### 7.3.3. Chemical Resistance

Numerous factors exert detrimental influences on the chemical composition of aligners, with notable culprits including saliva, temperature fluctuations, and a variety of enzymes [36]. In the oral environment, the absorption of moisture can lead to the degradation of the polymer’s molecular structure, resulting in a gradual reduction in the effective orthodontic forces they can exert over time [47]. In vitro investigations have demonstrated that the chemical constitution of polymers significantly impacts the tensile yield stress of aligners [9]. To effectively mitigate this degradation, it is imperative to gain a comprehensive understanding of the diverse chemical mechanisms governing structural alterations in polymer macromolecules, the reaction pathways of polymer additives, polymer morphology, and the intricate processes involved in oxidative chemistry [91]. Furthermore, intraorally, aligner materials often exhibit signs of wear, delamination, integument adsorption, and localized deposits of calcified biofilm at stagnant sites. These phenomena collectively contribute to reduced stiffness, heightened brittleness, and an increased susceptibility to crack formation in aligners [47]. Consequently, it has become evident that polymers chosen for clear aligners must possess resistance to hydrolysis and resist degradation caused by water exposure [36]. 

### 7.4. Optical Properties

Esthetic characteristics are a key factor for any orthodontic appliance, including 3D-printed devices [75]. It is important that patients do not feel social discomfort, as that may affect their compliance [87]. In light of the patient’s esthetic, the transparency of an aligner should remain stable for the 1–2 weeks of treatment, allowing the transmission of at least 80% of visible light [25,36]. Because transparency is a defining factor in clear aligners, amorphous polymers are more frequently used, as crystallinity tends to lead to opaqueness [36]. Aligners can also be stained from consuming-colored drinks, UV exposure, and the use of mouthwash [25,36,87]. Dentists will always recommend that patients remove their aligners before eating or drinking (except water), but this is not always followed, which can undermine transparency [25]. In addition, microcracks, delamination, and calcified biofilm deposits can lead to the loss of transparency [36]. Therefore, understanding these phenomena is essential and requires considering both the material properties as well as specifics of the 3D printing process. 

Typically, microcracks often occur due to the inherent brittleness of certain resin-based materials. The layer-by-layer printing process can introduce stress concentrations at the interfaces between layers that cause microcracks. Furthermore, the shrinkage of material during curing can create internal stresses. Materials with higher modulus of elasticity are generally more prone to microcracking [112,113]. 

Delamination refers to the separation of layers in a 3D printed object. It can occur due to inadequate bonding between layers, often a result of improper curing or temperature control during the printing process. Materials that require precise curing conditions, like certain methacrylate-based resins, can be susceptible to delamination if the process parameters are not optimally controlled [114].

Calcified biofilm deposits are related to the oral environment rather than the printing process. Bacteria in the mouth can adhere to the aligner surfaces, leading to the formation of biofilm. Over time, this biofilm can calcify, especially if the surface of the aligner is rough or porous. Materials with higher surface roughness or porosity can harbor more bacteria, leading to increased biofilm formation. Some resins may have surface characteristics that promote biofilm adherence [115,116].

To prevent these losses in transparency, materials should be designed with increased strength to prevent microcracking, proper curing to prevent delamination, and optimize model to prevent surface roughness or porosity that can harbor bacteria causing biofilm formation. 

### 7.5. Biocompatibility

Biocompatibility is commonly defined as the material’s ability to interact favorably with the host in a specific application, and this principle holds paramount significance in orthodontic treatment [117]. Ensuring the appliance used in orthodontics is biocompatible is of utmost importance, as it should not induce harmful cytotoxic or estrogenic effects on the patient [118]. The relatively short lifespan of aligners, typically lasting 1–2 weeks, provides a degree of protection against long-term degradation effects. However, the frequent replacement of aligners necessitates rigorous biocompatibility testing due to the potential introduction of new chemical substances with each new set [62]. Furthermore, clear aligners must maintain consistent biocompatibility and environmental stability over time to prevent any adverse interactions between cells and the material [11,27,119]. The types of biocompatibility tests to ensure the safety of aligners as a surface contacting medical device are explained in ISO 10993-01 [120] and ISO 7405 [121]. Generally, it includes cytotoxicity, acute toxicity, irritation, sensitization, and genotoxicity. 

While concerns exist regarding the biocompatibility and safety of 3D printed orthodontic aligners, the overall evidence suggests they are generally safe for most individuals when made with biocompatible materials and used appropriately. Studies by Scherer et al. [122] and Costa et al. [123] indicate that most resins utilized in aligner fabrication comply with biocompatibility standards (ISO 10993-01 and ISO 7405 [121]). However, potential cytotoxicity, allergic reactions to specific components, and residual monomer concerns have been identified in certain materials or under prolonged exposure conditions not typically encountered in standard treatment, as demonstrated by Li et al. [124]. 

It is important to note that all UV-curable resins are composed of monomers that can pose health risks in their non-polymerized states. For instance, even though acrylates and methacrylates are commonly used in 3D printing, they can cause skin irritation and other allergic reactions. Specifically, methacrylate-based monomers commonly used in these resins are known for skin irritation and sensitization risks. The degree of polymerization during the curing process affects the residual monomer content, as incomplete polymerization can leave residual monomers [125]. To ensure these negative effects are avoided, the degree of polymerization must be controlled. If residual monomers are present after the initial polymerization, further post-processing must take place to reduce the presence of the toxic monomers or dangerous materials. Special sterilization techniques can be used to further ensure the available surface of aligners are rid of bacteria or biofilm formation. For these reasons, it is important to conduct extensive cytotoxicity and biocompatibility studies prior to releasing 3D printing materials to be used in the body. The selection of materials and post-processing techniques is integral to creating a high-quality product with minimal adverse effects to the human body.

Choosing aligners from reputable manufacturers adhering to good manufacturing practices, coupled with patient-specific considerations and close dental supervision, as emphasized by Jeng et al. [126], are crucial for mitigating risks and ensuring safe and effective aligner treatment.

#### 7.5.1. Material Selection

The path to enhancing biocompatibility starts with careful material selection, where the most commonly employed base resins include acrylate or methacrylate monomers, blended with photoinitiators and any essential additives [11]. However, it is worth noting that methacrylate materials utilized in photopolymerization processes have been associated with partial toxicity concerns [11,118]. Prolonged exposure to the intraoral environment can impact the structural integrity of various material properties, such as hydrolytic stability and plasticization, potentially leading to the release of component molecules, most notably bisphenol-A (BPA) [127,128]. BPA, when released from certain plastics, is recognized as a potent endocrine disruptor capable of interfering with hormonal interactions in the body [62,118]. Moreover, it has been linked to conditions such as type-II diabetes, obesity, growth inhibition, behavioral changes, cardiovascular disease, and specific types of cancer [62]. Some current photocuring resins may also contain lipid-soluble heavy metal antimony compounds, known to provoke skin and mucosal irritation, or isocyanate [62,76]. Therefore, it is imperative to ensure that newly developed photocurable resins intended for medical applications exhibit no skin reactivity, carcinogenicity, or reproductive toxicity [76].

A study by Willi et al. [118] examining 3D-printed aligners found that the resin used in their production showed a high conversion rate and did not release bisphenol-A (BPA). However, the study detected significant amounts of urethane dimethacrylate (UDMA) in water eluents, with levels as high as 100 μg/L. This raised concerns about potential biological reactivity. Given that aligners are regularly replaced during orthodontic treatment, patients might be exposed to continuously high toxic levels of UDMA over several months. Furthermore, the study warns that the identified levels of UDMA might actually be an underestimation of the clinical situation due to various factors that can alter the material’s behavior.

Alternatively, epoxy acrylates are also considered to be an alternative option for the UV-curable resins. While epoxy acrylates have high crosslink density making them more rigid, they are far more reactive and can potentially lead to a higher percentage of unreacted epoxide groups as compared to urethanes. Furthermore, UDMA degradation products are less toxic and less likely to cause skin sensitization as well as allergic reactions than their epoxy counterparts due to the nature of their chemical bonds [129]. Henceforth, in dental applications that generally require direct and prolonged contact with biological tissues, UDMA is more favor due to its lower toxicity and reactivity.

To ensure the suitability of 3D printed dental models for intraoral applications, it is important that the resins undergo a rigorous testing regimen encompassing biocompatibility and cytotoxicity assessments, along with the attainment of FDA approval. These approved materials are additionally acknowledged for their exceptional dimensional stability, resistance to color alteration, and their ability to withstand degradation within the oral environment [21]. Moreover, maintaining consistent quality and averting contamination with non-intraoral resins necessitates the establishment of a meticulous and systematic procedure for washing and post-curing newly printed intraoral resins.

#### 7.5.2. Washing and Post-Curing

Ensuring the biocompatibility of 3D-printed dental models hinges significantly on two critical steps: thorough washing and meticulous post-curing processes [11]. Given that full polymerization may not be achieved during the 3D printing phase, post-curing becomes imperative to attain an optimal degree of conversion for the double bonds present in the methacrylic group. This conversion is accomplished through exposure to light and heat, typically in a UV cure box or furnace [111]. It is essential to recognize that while proper post-processing enhances the performance of printed samples, it does introduce additional demands in terms of time and cost [4].

Washing a denture resin with both an isopropyl alcohol and ether solution improves its biocompatibility without compromising its mechanical properties [11]. As part of the cleaning procedure, it is found that utilizing a centrifuge operating at 500 rpm for 5 min is able to adequately remove residual surface monomers from the dental models. However, it is important that they are positioned such that the cleaning agent can flush out the interior of the aligner during centrifugation [62,71]. It is found that extended post-rinsing times with isopropyl alcohol (IPA) lead to decreased flexural strength of Dental LT Clear Resin (Formlabs Inc., Somerville, MA, USA) [75].

In a study focused on clear dental aligners, researchers investigated different post-curing conditions and their impact on mechanical strength by subjecting the aligners to mechanical compression loading. The findings highlighted the essential role of post-cure processing in achieving necessary mechanical strengths and emphasized the need for well-defined specifications regarding post-curing time and temperature [130]. Additionally, heat treatment was observed to enhance the degree of cure on UDMA-based materials. The study by Andjela et al. [11] demonstrated that optimal properties were achieved when subjecting the aligners to a post-curing time of 10 min under UV light. Furthermore, higher post-curing temperatures (e.g., 60 °C and 80 °C) were found to improve the biocompatibility of dental resin. Interestingly, the mechanical properties of the printed dental aligners, such as flexural, tensile, and compressive strength, continued to improve until the 7th day after completing all curing steps, indicating that the polymerization process continued even after post-curing, leading to enhanced mechanical properties [11].

It is widely recognized that all resins contain toxic and allergic properties before 3D printing and UV curing [71], due to incomplete conversion of monomers into polymers during the printing process which causes a significant decrease in the degree of conversion, resulting in the release of potentially harmful monomers which their degradation and metabolization of these released monomers have been associated with irretrievable damage to cellular DNA [128]. Proper polymerization during the printing and UV curing stages is essential for enhancing biocompatibility and reducing the risk of adverse reactions to the patient [62].

#### 7.5.3. Antimicrobial Properties

Antimicrobial properties are essential in the design of clear aligners because insufficient oral hygiene bacteria form biofilms on the oral surfaces that can cause further oral health complications. Several studies were conducted to determine a relationship between the manufacturing method and surface quality on microbial biofilm formation and adhesion. It was found that manufacturing methods and printing resolution did not affect microbial adhesion substantially [11]. Printed aligners are new appliances that need to be tested both in vitro and in vivo [62]. In vitro studies placed an emphasis on the biocompatibility of the aligners, focusing on the cytotoxicity of the materials used within the dental field [25,127]. Common cytotoxicity tests include the use of a cell counting kit-8 (CCK-8) assay, hemolysis experiment, and LDH tests [76,119]. These tests are performed using mouse fibroblasts (L929 cells), human gingival fibroblasts (HGFs), and human lung fibroblasts (MRC-5) [11]. Using these tests on real cells allows for a simulation of how cells would react to the aligner when it is in use within the mouth. HGFs in particular are recommended by ISO because they constitute the main cell line present in the oral tissues and are the most exposed to the toxic effects of the materials used in aligners [25]. 

In a study by Pratsinis et al. [127], cytotoxicity, antioxidative activity, and estrogenicity of Tera Harz TC85A resin printed on a SprintRay Pro 55 printer (SprintRay, Los Angeles, CA, USA) were examined. No adverse effects on human gingival fibroblasts were observed after 2 weeks of exposure to the resin. However, this study did not consider environmental factors like chewing forces, thermal changes, or oral microbiota. Additionally, the antioxidant Trolox reduced reactive oxygen species (ROS) levels, and no xenoestrogenic activity was found. Rogers et al. [117] conducted another study exploring 3D printing materials for specialized long-term culture of reproductive cells and tissues. They tested two biocompatible resins and observed rapid degeneration of mammalian oocytes in vitro. The study raised concerns about the cytotoxicity of bisphenol A (BPA), a common plastic additive, and noted the absence of reproductive health safety testing in ISO biocompatibility certification unless there is direct contact with reproductive tissues.

To diminish residual uncured resin in 3D printing materials, one effective method involves integrating a high-molecular cationic polymer within a semi-interpenetrating network. This innovative approach not only exhibits antimicrobial capabilities but also inhibits the formation of a salivary conditioning film, as highlighted in recent research [11]. Alternatively, the utilization of zwitterionic materials has shown promise in bolstering antimicrobial properties by leveraging electrostatic interactions to deter protein adhesion and biofilm formation. However, it’s worth noting that this strategy may lead to a reduction in mechanical properties [11]. Remarkably, there remains a notable gap in research when it comes to investigating the cytotoxic and estrogenic effects associated with 3D printing resins and products [71].

## 8. Current Challenges of the 3D Printed Aligners

**Figure 5:**
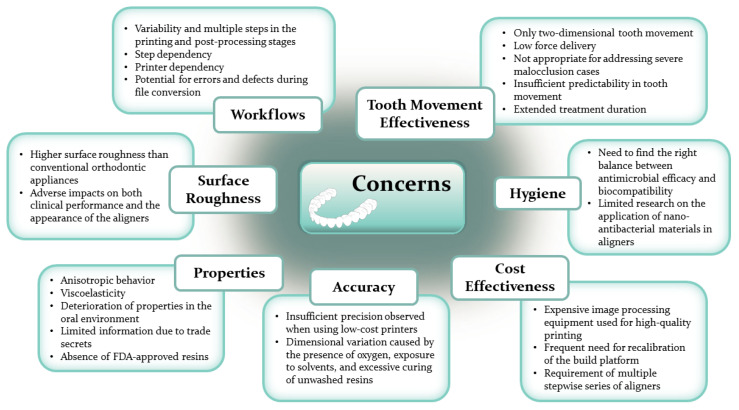
Challenges in the 3D-printed clear aligners.

The main challenges in 3D printing aligners stem from the absence of a material meeting all necessary criteria like biocompatibility, translucency, 3D printability, and appropriate mechanical properties [42]. Prior to clinical use, several issues must be tackled, falling into categories such as (1) workflows, (2) anisotropic behavior, (3) properties, (4) accuracy, (5) affordability, (6) tooth movement effectiveness, and (7) hygiene of 3D printed clear aligners (Figure 5).

### 8.1. Workflows

The initial challenge pertains to workflows. Typically, printing and post-processing involve inconsistent, multi-step procedures [62]. Errors in any step can adversely impact subsequent stages, resulting in deviations in the final product. For instance, during aligner printing, trapped porosity between material layers can reduce interfacial adhesion, affecting overall mechanical properties [1]. Additionally, printed aligner quality varies by printer, as different irradiation exposure conditions and CAD conversion processes lead to differing outcomes [62,71].

### 8.2. Surface Roughness

The second challenge arises from the surface roughness of 3D-printed aligners, typically ranging from 0.87 to 4.44 µm, which exceeds that of conventional aligners, cast, and milled structures [71,90]. High surface roughness results from incomplete UV curing and intraoral aging, triggering the release of substances in the oral cavity. This roughness can lead to small fractures in the aligner, compromising mechanical properties, clinical effectiveness, and safety, as well as causing a foggy appearance due to reduced light transmission. Surface roughness is influenced by printing orientation, with vertical printing creating more layers and roughness, while horizontal printing produces smoother surfaces. Most 3D printers have a resolution of around 50 m, yielding less noticeable layer lines and smoother surfaces but requiring longer printing times. The impact of aligner layer thickness (50 m vs. 100 m) on treatment outcomes remains unclear. Residual uncured resin can also contribute to increased surface roughness, underscoring the importance of thorough cleaning before post-processing [71].

### 8.3. Properties of Aligners

The third challenge pertains to obtaining the appropriate properties for aligners. 3D printing materials exhibit anisotropic behavior, which varies depending on part geometry, printing orientation, print settings, and temperature, resulting in different mechanical properties under various loading conditions [1,17]. Additionally, aligners possess viscoelastic properties and are designed to apply low force, work effectively with minimal activation, exhibit low flexibility, and experience rapid force decay [47]. Unlike traditional archwires and brackets, oral conditions, such as temperature, humidity, and salivary enzymes, adversely affect aligner mechanical behavior and dimensional stability over time. This is due to the degradation of the aligner’s polymer molecular structure through water absorption and the deposition of calcified biofilm in the oral environment, leading to reduced elastic modulus, increased brittleness, and crack development [10,47,76]. Despite multiple studies investigating aligner mechanical properties, comparisons are challenging due to differences in materials, setting parameters, and printing processes used, which is a major limitation [11].

Several constraints are evident in the field of dental resins for 3D-printed aligners. One significant limitation is the lack of information on the chemical composition of existing dental resins, mainly due to trade secrets [11]. Additionally, there is a shortage of Food and Drug Administration (FDA)-approved resins for direct 3D-printed aligners, although several companies are actively involved in advanced development stages [21]. Moreover, the limited number of materials tested for biocompatibility hampers the use of 3D-printed photopolymer resins in dental device fabrication [11,26,131], further impeding progress in dental treatments utilizing 3D printing technology [19].

### 8.4. Accuracy

The fourth challenge concerns the accuracy of the final product. Low-budget, entry-level 3D printers used to create orthodontic models for clear aligners may encounter accuracy issues, as reported in various studies [19]. For example, commercially available aligners have shown gap volumes ranging from 107 to 402 mm [87]. Evaluations of 3D-printed orthodontic models have mostly focused on aligned teeth, which differs from real-world clinical scenarios where teeth can be misaligned and crowded. This crowding factor can significantly affect the precision and diagnostic reliability of 3D-printed models [19].

Additionally, in the established workflow, freshly printed aligners require further polymerization in UV curing units. However, the presence of oxygen in these units hinders complete resin polymerization [62,71]. This challenge is exacerbated by the variable interfacial space of approximately 11 μm between each 3D-printed layer, leading to uneven photopolymerization due to oxygen diffusion over time and potential sample expansion with extended follow-up periods [90]. To enhance the quality of printed aligners in terms of mechanical properties, leaching, and aging, a solution involves printing them in an oxygen-free environment using a curing chamber equipped with nitrogen gas [71]. Additionally, despite photopolymers’ inherent solvent resistance, they are prone to swelling, leading to volume increases and dimensional deviations from the initial design [90].

Another concern related to the accuracy of 3D-printed aligners is associated with the translucent nature of the resin and the potential lack of sufficient photo absorber additives. If the freshly printed aligner is not thoroughly washed before post-polymerization, excess resin may accumulate in the depressed areas of the aligner design and cure during printing [89]. Despite suggested processing conditions in IPA baths, some residual excess resin may settle in the central grooves of the posterior teeth and cure during post-curing, resulting in thicker specimens [73].

### 8.5. Cost Effectiveness 

The fifth challenge concerns the cost and handling difficulties associated with current 3D printing technologies [1,62]. This issue stems from the early stages of digital dentistry, where acquiring imaging data and printing equipment is both costly and challenging [6]. While the translucency of the material used in clear aligners is advantageous for aesthetics, it presents challenges during scanning, requiring the application of a contrast spray for optical scanner registration [42].

Additionally, the novelty of 3D printing technologies, without complete optimization, leads to high processing costs and time-consuming post-processing [4]. There’s a need to optimize printing speed and post-processing to meet dental, technical, and biocompatibility standards [7]. However, the aligner production process remains time-consuming and demands a heavy laboratory workload [55]. Entry-level 3D printers often require frequent recalibration of the build platform due to printing failures, potentially affecting in-office CAD/CAM workflow efficiency, especially in treatments involving numerous aligners [19].

There are limited studies describing direct 3D printing of clear aligners [10], and it’s important to note that the material is not currently marketed specifically for direct aligner fabrication [42]. The stepwise staging of aligners consumes significant time and materials, resulting in higher treatment costs primarily due to the limited movement achieved by each individual aligner [78].

### 8.6. Tooth Movement Effectiveness 

The sixth challenge pertains to the effectiveness of tooth movement with clear aligners. While clear aligners can achieve certain tooth movements and alignment [27,63], they face difficulties in torque control [27]. These aligners primarily utilize pushing forces, requiring attachment surfaces for tooth extrusion. Unlike fixed appliances that allow for three-dimensional tooth movement, clear aligners predominantly focus on two-dimensional movement, resulting in more tipping than root movement [55]. Transparent aligners apply continuous, relatively low force compared to fixed appliances, leading to reduced tension and compression for lingual inclination and axial rotation deformation [38]. Several studies have demonstrated that clear aligners are less effective than traditional fixed appliances in addressing extrusive movements, rotations of rounded teeth, and buccolingual inclination of anterior teeth [50,55,63,72,132]. Additionally, they have limited efficacy in treating intrusion and overbites [132].

Aligners exhibit a stiffness 40–50 times lower than a typical NiTi archwire, making them easily deformable with minimal force and less resistant to permanent deformation compared to archwires. This characteristic necessitates the use of a series of aligners for correcting even minor crowding, rather than a single NiTi archwire [47]. The complexity of tooth movement with aligners arises from factors like the absence of specific force application points, tooth anatomy, aligner material properties, geometric differences, slipping motions, and other biomechanical factors, which pose challenges for accurate treatment prediction [47]. An ideal aligner should deliver a light, constant force over time, be stiff with high-yield strength, and tolerate varying degrees of deformation to create the desired force system. Stiff materials with high elastic modulus can generate more effective force systems with minimal deformation, while flexible materials can easily deform [47].

As discussed in the study by Seo et al. [38], transparent aligners experienced a significant reduction in force over time, with a 50% and 75% decrease after 8 h and 4 days, respectively. Aligners have low resilience, as they mostly dissipate energy as heat rather than transferring it to teeth effectively [47]. They are more suitable for mild to moderate malocclusion cases, and their effectiveness is limited in extreme malocclusions [44,48,133]. Difficulties are encountered in achieving tooth extrusion, canine and premolar rotation, bodily movements, and root torque [44]. Researchers have determined that clear aligners are not well-suited for extreme malocclusions involving significant crowding or spacing, substantial skeletal anterior-posterior differences, highly rotated teeth, anterior and posterior open bite, severely tipped teeth, teeth with short crowns, jaw realignment, and arches with multiple missing teeth [48,133].

Clear aligners have limited reliability when it comes to movements beyond minor horizontal shifts and struggle to accurately predict rotational tooth movements, especially for canines. While they can handle tipping movements, they struggle with controlling root movements due to the applied stress levels [9,47,61,63]. Despite technological advancements, clear aligners generally lack predictability in tooth movement, potentially resulting in differences between the intended final occlusion and the actual outcome [9,63].

Compared to fixed appliances, transparent aligners place less stress on the alveolar bone during tooth movement, resulting in longer orthodontic treatment periods [38]. The prevailing consensus acknowledges a significant discrepancy between predicted and clinical outcomes, often necessitating multiple refinement stages or additional treatment [47]. This extended treatment period can be attributed to the lower force applied by the polymer materials in clear aligners compared to the metal used in fixed appliances, leading to reduced tooth movement [38]. Furthermore, research suggests that the effectiveness of treatment plans using clear aligners may decrease with elevated patient age [63].

### 8.7. Hygiene 

The final challenge involves aligner hygiene, requiring a balance between antimicrobial effectiveness and biocompatibility. One intriguing solution on the horizon involves incorporating bioactive glass as a filler, releasing helpful ions, establishing strong bonds with hard tissues, and addressing the issue of polymerization shrinkage [11]. Nevertheless, as of now, there are only a few research studies investigating the use of nano-antibacterial materials in orthodontic aligners [36]. Consequently, it is imperative that we dedicate further research efforts to explore this promising area.

## 9. Future Direction of the 3D Printing Aligners

The field of clear aligner therapy has advanced significantly, expanding its use beyond mild to moderate cases to a broader range of malocclusions. These improvements resulted from innovative treatment approaches [44,61]. To improve aligner retention, two critical factors must be considered: the use of attachments and power ridges [47,84], and careful selection of the aligner material [84].

Attachments play a vital role in clear aligner techniques, providing torque, tooth rotation, and influencing treatment outcomes when compared to the virtual setup [27,87]. They guide orthodontic movements effectively through moment-to-force ratio components, enabling precise force application to specific teeth for enhanced predictability [47,61,133,134]. Correct positioning is crucial for effective movement, and power arms have been introduced to optimize biomechanics by aligning the force vector closer to the tooth’s center of resistance [86]. Incorporating these advancements leads to improved orthodontic treatment outcomes.

Material selection significantly impacts retention [84]. Acrylates in the composition lead to faster cross-linking reactions and less oxygen inhibition. Cycloaliphatic and aromatic acrylates exhibit reduced shrinkage compared to standard monomers [111]. Aligner tooth movement depends on shape changes, and discrepancies can create the pushing force for crown tip movement [84]. Enhancing composite attachments’ mechanical properties improves aligner surface contact and complements aligner efficacy [43,135]. Additionally, multi-component systems containing various properties as well as material components can be utilized to tailor desired properties and customize movement of the direct 3D printed clear aligners [136,137]. 

Moreover, optimizing the 3D-printed aligner manufacturing, especially post-processing techniques, are crucial alongside attachments and material selection. Aligners exert substantial force on teeth, relying on thorough UV curing for their potential properties to shine. Complete polymerization is essential for aligner transparency, reduced roughness after aging, and enhanced mechanical properties [62]. Challenges like thermal deformation, material shrinkage, and expansion can lead to thickness variations, affecting the aligner’s final outcome [62,73].

Furthermore, integrating artificial intelligence (AI) into dentistry offers promising opportunities to enhance aligner manufacturing through 3D printing and treatment outcomes. The current model for simulating orthodontic movement with aligners lacks clinical accuracy [75]. Future studies should focus on transparent aligners with attachments and finite element analysis for severe-crowding dentition [38]. AI integration can predict growth and optimize treatment design, improving outcomes by automating diagnosis, prediction, and appliance design, reducing human biases, and standardizing processes [41]. Dentistry can lead the way in implementing AI for routine tasks and functions by harnessing vast data to evaluate treatment effectiveness [138].

Finally, the integration of 4D printing in dentistry holds the promise of introducing precision and innovation into treatment methodologies. 4D printing harnesses the power of intelligent materials to fashion structures capable of changing shape in response to external stimuli, such as water, heat, light, or electricity, evolving over time [71,139]. This transformative technology finds applications not only in fields like stem cell research and tissue engineering but also in dentistry, where it elevates the precision and retention of prosthetic devices [139]. In addition, the harmonious blend of 4D printing with artificial intelligence (AI) portends dynamic, patient-specific treatment approaches, ushering in improvements in prosthetic fitting, patient outcomes, and overall quality of life.

## 10. Conclusions

Clear aligners have emerged as a highly valuable and lucrative tool in the dental industry, resulting in a significantly high-value market. Over time, manufacturing methods have evolved from vacuum forming to 3D printing, offering numerous advantages such as complex geometrical structures, faster production, customized designs for patients, reduced material and labor costs. Different 3D printing methods have found diverse applications in the dental field, ranging from learning tools to various dental specialties like oral surgery, prosthodontics, periodontics, pediatric dentistry, oral implantology, and orthodontics. These applications have significantly enhanced the quality of patient care, reduced waiting times, and triggered further research and improvements in material properties and biocompatibility. The advancements in 3D printing have led to less invasive and cost-effective aligner treatments, while maintaining their effectiveness compared to conventional treatment options. Nonetheless, there remains potential for further research in enhancing tooth movement effectiveness, focusing on areas such as aligner retention, improved attachments, and enhanced processing techniques. As the dental industry progresses toward 4D printing, the future promises even more effective treatments and improved quality of life for patients. With the continued implementation of additive manufacturing, clear aligners are poised to revolutionize the treatment of minor malocclusions and positively impact the lives of numerous future patients.

## Figures and Tables

**Table 1:** Comparison of various 3D printing techniques in dentistry.

3D Printing Techniques	Materials	Advantages	Disadvantages	References
Fused Deposition Modeling (FDM)	- Low-melting point polymers	- Simple and low-cost printing - Short processing time - Clean printing process	- Low printing accuracy and precision - Poor surface finish parts - Supporting structure required	[17,31,32]
Selective Laser Sintering/Melting (SLS/SLM) and Powder Bed Fusion (PBF)	- Metals - Ceramics - High-melting point polymers	- No requirement for supporting materials - Parts produced with high mechanical and physical properties	- Possibility for structural damage from a high-energy laser - Poor surface finish parts - High equipment cost - Shrinkage and warping products	[17,31,32]
Polyjet	- Photopolymers - Waxes	- Ability to spray various materials simultaneously, generating a product with the desired color and mechanical properties - High precision and accuracy printing - Low printing layer thickness - Ability to produce complex geometrical shapes - Smooth surface finish	- Only suitable for a short-term usage - Expensive materials and equipment - High material consumption - Long printing duration - Not suitable for a large-scale production	[12,17,31,32,33,34]
Stereolithography (SLA)	- Photopolymers - Ceramics	- High printing speed - High-temperature resistance - Ability to generate complicated geometric parts - High printing accuracy - Smooth surface finish	- High material consumption due to a requirement for supporting structures - High equipment cost - Lower printing precision than the DLP and polyjet method - Not suitable for a large-scale production - Post-processing required	[12,17,31,32,33,34,35]
Digital Light Processing (DLP)	- Photopolymers - Ceramics	- High printing accuracy and precision - High printing speed - Suitable for generating complex geometrical parts - Consistence printing layer thickness - Smooth surface finish	- Supporting structure required - Post-processing required	[12,17,32,33,34,35]

## Data Availability

Not applicable.
